# Amino Acid Profile in Malnourished Patients with Liver Cirrhosis and Its Modification with Oral Nutritional Supplements: Implications on Minimal Hepatic Encephalopathy

**DOI:** 10.3390/nu13113764

**Published:** 2021-10-25

**Authors:** Silvia Espina, Yolanda Gonzalez-Irazabal, Alejandro Sanz-Paris, Marta Lopez-Yus, Maria Pilar Garcia-Sobreviela, Raquel del Moral-Bergos, Beatriz Garcia-Rodriguez, Javier Fuentes-Olmo, Vanesa Bernal-Monterde, Jose M. Arbones-Mainar

**Affiliations:** 1 Gastroenterology Department, University Hospital Miguel Servet, 50009 Zaragoza, Spain; silespina@gmail.com (S.E.); fuentesolmo@gmail.com (J.F.-O.); vbernalm@gmail.com (V.B.-M.); 2 Instituto de Investigación Sanitaria (IIS) Aragon, 50009 Zaragoza, Spain; yolgonira@gmail.com (Y.G.-I.); sanzparisalejandro@gmail.com (A.S.-P.); martalyus@gmail.com (M.L.-Y.); mpgarciaso.adipofat@gmail.com (M.P.G.-S.); rdelmoral.iacs@aragon.es (R.d.M.-B.); bea_garcia_rodriguez@hotmail.com (B.G.-R.); 3 Clinical Biochemistry Department, University Hospital Miguel Servet, 50009 Zaragoza, Spain; 4 Nutrition Department, University Hospital Miguel Servet, 50009 Zaragoza, Spain; 5 Adipocyte and Fat Biology Laboratory (AdipoFat), Translational Research Unit, Instituto Aragones de Ciencias de la Salud (IACS), University Hospital Miguel Servet, 50009 Zaragoza, Spain; 6 Centro de Investigación Biomédica en Red Fisiopatología Obesidad y Nutrición (CIBERObn), 28029 Madrid, Spain

**Keywords:** HMB, hydroxymethylbutyrate, clinical trial, PCA

## Abstract

Low plasma levels of branched chain amino acids (BCAA) in liver cirrhosis are associated with hepatic encephalopathy (HE). We aimed to identify a metabolic signature of minimal hepatic encephalopathy (MHE) in malnourished cirrhotic patients and evaluate its modification with oral nutritional supplements (ONS) enriched with ß-Hydroxy-ß-methylbutyrate (HMB), a derivative of the BCAA leucine. Post hoc analysis was conducted on a double-blind placebo-controlled trial of 43 individuals with cirrhosis and malnutrition, who were randomized to receive, for 12 weeks, oral supplementation twice a day with either 220 mL of Ensure^®^ Plus Advance (HMB group, *n* = 22) or with 220 mL of Ensure^®^ Plus High Protein (HP group, *n* = 21). MHE evaluation was by psychometric hepatic encephalopathy score (PHES). Compared to the HP group, an HMB-specific treatment effect led to a larger increase in Val, Leu, Phe, Trp and BCAA fasting plasma levels. Both treatments increased Fischer’s ratio and urea without an increase in Gln or ammonia fasting plasma levels. MHE was associated with a reduced total plasma amino acid concentration, a reduced BCAA and Fischer´s ratio, and an increased Gln/Glu ratio. HMB-enriched ONS increased Fischer´s ratio without varying Gln or ammonia plasma levels in liver cirrhosis and malnutrition, a protective amino acid profile that can help prevent MHE.

## 1. Introduction

Malnutrition in cirrhosis is characterized by an increase in fatty acid oxidation, reduced utilization of glucose as energy source, and decreased protein synthesis. All these processes converge into a loss of muscle mass [1]. Gluconeogenesis is increased in early stages of liver cirrhosis and the skeletal muscle is the main source of amino acids by proteolysis. As a result, both aromatic (AAA) and branched chain amino acids (BCAA) are generated [2]. BCAA are catabolized in the skeletal muscle and AAA are primarily metabolized in the liver. As a consequence, plasma BCAA concentrations and Fischer´s ratio (BCAA/AAA) are reduced in cirrhotic patients [2,3,4]. The reduced concentration of amino acids in the muscle cells activates proteolysis via autophagy [2]. Hyperammonemia plays a crucial role in increased BCAA catabolism and decreased plasma BCAA concentrations in liver cirrhosis [5]. Moreover, hyperammonemia in cirrhosis also contributes to the inhibition of protein synthesis and the activation of autophagy [2,6].

Physiologically, glutamate (Glu) serves as an anaplerotic substrate in most tissues to generate α-ketoglutarate, and this reaction is catalyzed bidirectionally by the enzyme glutamate dehydrogenase [2]. However, in cirrhosis due to impaired ureagenesis, the skeletal muscle is the main ammonia-detoxifying system by increasing the activity of glutamine synthetase [2,7]. As consequence, Glu and ammonia form glutamine (Gln) and consume α-ketoglutarate [8]. Most of the Gln produced is released to the blood and catabolized in enterocytes and kidneys to ammonia [9]. This Gln synthesis and Gln breakdown in liver cirrhosis plays a vicious cycle in the development of hyperammonemia and in the decrease in BCAA levels [10]. Ultimately, increased blood ammonia levels causes astroglial swelling leading to neurological or psychiatric alterations known as hepatic encephalopathy (HE) [11]. In sarcopenia, due to low muscle mass, non-hepatic disposal of ammonia is impaired, which may cause HE more frequently [12]. HE is divided into two broad categories based on severity, covert or minimal (MHE) and overt [13]. MHE has a significant impact on a patient´s quality of life, increasing hospitalizations and death [14]. The psychometric hepatic encephalopathy score (PHES) is a validated, gold standard tool to evaluate MHE [15].

Supplementation with leucine (Leu), a BCAA, actives protein synthesis via mTOR (mammalian target of rapamycin) [16]. Moreover, BCAA supplementation promotes ammonia detoxification in skeletal muscle via α-ketoglutarate and serves as an anaplerotic substrate in muscle and brain [17]. Clinically, BCAAs in liver cirrhosis have been associated with symptomatic improvement of hepatic encephalopathy and decreased incidence of ascites and infections [18]. An additional increase in muscle strength has also been observed [18]. From studies of amino acid oxidation [19], it has been advised that BCAA supplementation in liver cirrhosis be carried out in the proportions 2:1:1 (Leu:Ile:Val) [20]. Supplementing with leucine alone could increase the oxidation of other BCAAs, worsening muscle protein balance [21].

ß-Hydroxy-ß-methylbutyrate (HMB) is a naturally occurring and metabolically active derivative of the BCAA Leu [22]. HMB stimulates protein synthesis and inhibits the ubiquitin–proteasome system [23], and is more effective than Leu in increasing protein synthesis through the mTOR system [24]. HMB also increases the activity of branched-chain alpha-keto acid dehydrogenase (BCKD) in muscle-wasting disorders such as liver cirrhosis. This results in the oxidation of Leu as source of energy, increasing acetyl-CoA and impairing HMB production [7].

Recent studies have shown that 12-week HMB supplementation improved muscle performance in compensated cirrhotic patients [25] and increased plasma BCAA in cirrhotic and partially hepatectomized rats [26,27]. However, the effect of oral nutritional supplements (ONS) with HMB on the amino acid profile of patients with decompensated cirrhosis has not been studied. We therefore conducted a pilot randomized clinical trial aimed at analyzing the effect of HMB in the clinical setting of liver cirrhosis and malnutrition (Espina S. Unpublished results). In this post hoc study we aimed to identify a metabolic signature of MHE in malnourished cirrhotic patients and evaluate its modification with ONS.

## 2. Materials and Methods

### 2.1. Study Design and Population

This post hoc study is part of a double-blind, parallel-group, randomized controlled trial (NCT03285217 at ClinicalTrials.gov) performed in the University Hospital Miguel Servet (Zaragoza, Spain) that enrolled patients with liver cirrhosis of any etiology, with 1 previous decompensation, and clinical malnutrition screened by SGA (Subjective Global Assessment) [28]. Exclusion criteria were being <18 years old or having diabetes mellitus, corticosteroid treatment, hepatocellular carcinoma, uncontrolled infection, orthotopic liver transplantation, recent (<3 months) overt hepatic encephalopathy, or variceal upper gastrointestinal bleeding.

Participants were randomized into one of two study groups with a 1:1 ratio via permuted-block randomization with random block size using the package *blockrand* of R. Participants randomly received, twice a day and for 12 weeks, ONS of either 220 mL of Ensure^®^ Plus Advance (HMB group; 1.5 kcal/mL, 24.3% protein, 28.8% fat, and 1.5 g of calcium HMB per service) or 220 mL of Ensure^®^ Plus High Protein (HP group; 1.25 kcal/mL, 25.3% protein, 23.8% fat). Supplementary Table S1 shows the amino acid content of each oral supplement.

This trial was conducted in compliance with the Declaration of Helsinki and was approved by the local ethics committee (CEIC-A, ref. PI17/0258). All study participants provided written informed consent before participating in the trial.

Amino acid profiles, along with sex and age, from otherwise healthy unidentified noncirrhotic individuals were provided by the Clinical Biochemistry Department at the University Hospital Miguel Servet. No informed consent was sought from those individuals due to the use of anonymized data.

### 2.2. Outcome Measures

Both groups were evaluated at the same time points: baseline (pretreatment), 6 weeks after the start of the ONS, and at the end of the treatment (12 weeks). At each visit, laboratory (fasting venous sampling) and clinical assessments were carried out. MHE was evaluated using the psychometric hepatic encephalopathy score (PHES), a battery of paper-pencil tests evaluating cognitive and psychomotor processing speed as well as visuomotor coordination [15]. Liver status was assessed with (1) Child–Pugh score [29] which uses five clinical measures of liver disease (total bilirrubin, serum albumin, prothrombin time, ascites, and HE). Each measure was scored 1–3 according to well-established cutoffs, with 3 indicating most severe derangement. A further classification into classes A (5–6 points), B (7–9 points), and C (10–15 point) was carried out [30] and (2) MELD score [31], which is used to assess the severity of chronic liver diseases according to a formula that includes values for serum bilirubin, serum creatinine, and the international normalized ratio for prothrombin time (INR).

Amino acids in plasma were identified using cation-exchange chromatography by post-column ninhydrin derivatization on the Biochrom 30+ Amino Acid analyzer (Biochrom Ltd., Cambridge, UK). Five different lithium citrate elution buffers were used to separate the amino acids, and a postcolumn reaction with ninhydrin formed a dye complex with the amino acids. UV detection of amino acids was obtained at two wavelengths, 570 nm and 440 nm, according to the type of the amino acids determined [32,33]. BCAA was calculated as the sum of Leu, Ile and Val. AAA was calculated as the sum of Phe, Tyr and Trp. Fischer´s ratio was calculated as the sum of Leu, Ile and Val divided by the sum of Phe and Tyr. Gln/Glu ratio was calculated as Gln divided by Glu.

### 2.3. Statistical Analyses

Statistical analysis was carried out in R 3.4.3. and the appropriate packages according to the predefined statistical analysis plan. Categorical data were presented as number of cases and percentages and compared using the Chi-squared tests with Yates correction. Continuous data were described as median and interquartile ranges (IQRs) and comparisons were performed with Mann–Whitney U-tests.

Principal component analysis (PCA) was performed with the *FactoMineR* and *factoextra* packages using scaled data. Variable contributions for a given principal component were expressed in percentage.

Longitudinal variations were modeled using linear mixed-effects models (LMM) for repeated measures using the *lme* function of the *nlme* package to take into consideration the repeated assessment of each variable. Missing values in outcome variables were not imputed. LMM models produced different *p*-values that captured the variation over time of each variable for the entire cohort (*p_long_*) and treatment-specific longitudinal changes, that is, the interaction between longitudinal changes and treatment (p_long*treatment_). Values of p_long*treatment_ < 0.1 were considered as hypothesis-generating.

## 3. Results

### 3.1. Specific Plasma Amino Acid Profile in Cirrhotic Patients

Firstly, we sought to investigate the difference in plasma amino acid profile between cirrhotic patients with clinical malnutrition (*n* = 43) and healthy individuals (*n* = 31) matched for age and sex.

Patients with cirrhosis had lower plasma concentration of phosphoserine, Asn, Gln, Val, and cystine while those of taurine, Asp, Ser, Glu, aminoadipic acid, Gly, Met, Tyr, beta-alanine, Phe, etanolamine, ornithine, Arg, Trp, His, 3-methylhistidine, and hydroxyproline (Hyp) were significantly higher in cirrhotic patients than in their healthy counterparts (Table 1).

**Figure 1:**
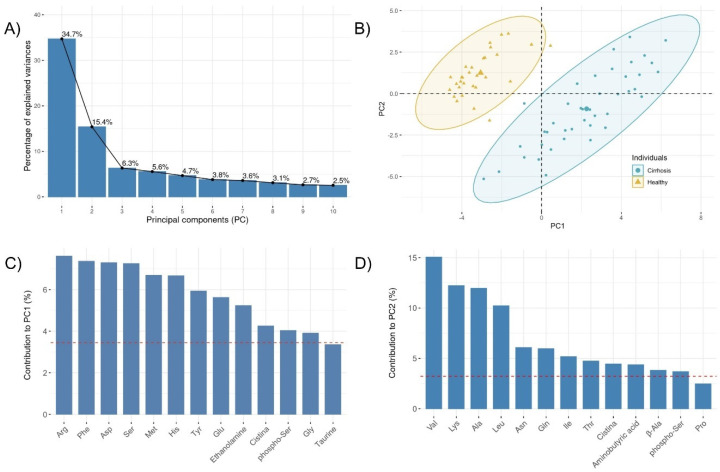
Principal Component Analysis (PCA). Scree plot with the proportion of information retained by each principal component (**A**). Biplot of individuals (**B**). The most contributing variables to the principal component 1 (**C**) and principal component 2 (**D**). The red dashed line indicates the expected average contribution.

Changes in the balance of the plasma amino acids were next investigated using principal component analysis (PCA). The Kaiser–Meyer–Olkin measure of sampling adequacy was 0.76 and the Bartlett´s test of sphericity rejected the null hypothesis of the test, i.e., that the variables were orthogonal (*p* < 0.001). Five PCs with eigenvalues >1 were identified and their percentage of explained variance is represented in Figure 1A. To evaluate their performance, the Mann–Whitney U-test was used to compare each PC score between the healthy and cirrhotic individuals. Both PC1 and PC2 showed significant *p* values (< 0.001) and their score plots showed a clear discrimination between cirrhotic and healthy subjects on the basis of their amino acid profile (Figure 1B). Next, we extracted the contribution of each individual amino acid towards the PCs. The results identified Arg, Phe, Asp, and Ser as the most contributing factors for PC1 (Figure 1C) while Val, Lys, Ala, and Leu were the most important contributors for PC2 (Figure 1D).

### 3.2. Baseline Characteristics of Cirrhotic Patients before Oral Nutritional Supplementation

Forty-three patients with cirrhosis were randomized to receive ONS twice a day with either 220 mL of Ensure^®^ Plus Advance (HMB group, *n* = 22) or oral supplementation twice a day with 220 mL of Ensure^®^ Plus High Protein (HP group, *n* = 21). At the time of enrollment, there were no significant differences between treatment groups with respect to age, sex or prevalence of ascites. Alcohol addiction was the main etiology of cirrhosis in both groups (Table 2).

Median [IQR] values for prognostic assessment in liver cirrhosis for all cirrhotic patients were 12 [8.5; 16.5] for the MELD score and 7 [6.0; 8.5] for the Child–Pugh score with no difference between treatment groups. The patients also underwent a nutritional assessment using the SGA scale without finding differences between the groups. Overall, 72% of the patients were evaluated as moderately malnourished (B) and the other 28% as severely malnourished (C) (Table 2).

A similar amino acid profile was observed in the treatment groups at the baseline with only minor differences in the plasma concentration of Leu and Trp (Supplementary Table S2). Both amino acids appeared slightly decreased at baseline in the HMB group.

### 3.3. Longitudinal Changes in Metabolic Parameters and Amino Acids during Oral Nutritional Supplementation

**Figure 2:**
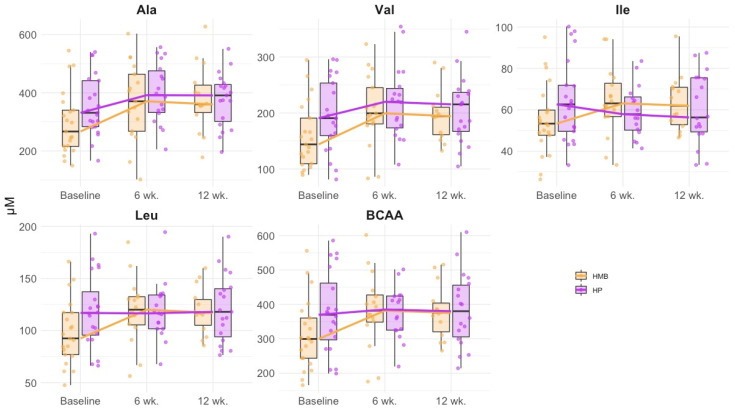
Plasma amino acid variations during follow-up in all cirrhotic patients according to treatments. HMB: HMB group, HP: High Protein groups. BCAA: branched chain amino acids. Each point represents a single subject. Boxes are drawn from Q1 to Q3 with a horizontal line drawn in the middle to denote the median.

A total of 108 amino acid profiles were assayed during the ONS treatment. Both ONS treatments during 12 weeks increased the plasma levels of Asp (*p_long_* = 0.001), Ala (*p_long_* = 0.004), citruline (*p_long_* = 0.03), Val (*p_long_* = 0.004), Met (*p_long_* = 0.03), Leu (*p_long_* = 0.01), Tyr (*p_long_* = 0.001), Phe (*p_long_* = 0.001), Lys (*p_long_* = 0.031), and Trp (*p_long_* = 0.008). Compared with the HP group, an HMB-specific treatment effect led to a larger increase in Val (35% vs. 13%, p_long*treatment_ = 0.055), Leu (27% vs. 1%, p_long*treatment_ = 0.035), Phe (36% vs. 0%, p_long*treatment_ = 0.057), and Trp (35% vs. 11%, p_long*treatment_ = 0.066) (Table 3). Changes observed in those amino acids are illustrated in Figure 2.

Plasma BCAA levels (sum of leucine, isoleucine and valine) increased significantly (*p_long_* = 0.012) during both treatments. However, this increase was larger in the HMB group compared with the HP group (25% vs. 3%, p_long*treatment_ = 0.046). AAA levels (sum of phenylalanine, tyrosine and tryptophan) increased significantly at the end of both treatments (*p_long_* = 0.003) without differences between treatment groups (Table 3). Supplementary Table S3 shows the variations for all plasma amino acid.

During follow-up the plasma urea levels increased significantly at the end of the trial (*p_long_* = 0.048), with no differences between treatments. Ammonia plasma levels did not increase significantly at the end of the trial (*p_long_* = 0.11).

### 3.4. Amino Acid Signature of the Minimal Hepatic Encephalopathy

Overall, 29% of the patients presented MHE during the baseline assessment while this percentage decreased to 21% after 12 weeks of ONS (*p* = 0.32). Interestingly, HMB treatment reduced the MHE prevalence from 38% to 21% (*p* = 0.16) while no reduction was observed in the HP group (19% in the baseline and 21% after treatment. *p* = 1).

108 amino acid profiles were obtained and 26 MHE events were documented during follow-up. Table 4 shows the metabolic profiles, closest in time to the MHE event, compared to those analyses that were not associated with any event. MHE events were associated with reduced total plasma amino acid concentration (calculated as the sum of all amino acid levels); 2.9 mM [2.6; 3.2] vs. 3.2 mM [2.9; 3.6] in the absence of MHE (*p* = 0.011). For the individual amino acids, significant lower plasma levels of taurine, Asp, Ser, Glu, Gly, Ala, Val, Ile, Leu, Phe, and Arg were associated with MHE. In general, a reduced BCAA and Fischer´s ratio and an increased Gln/Glu ratio occurred simultaneously during the MHE events.

## 4. Discussion

Portosystemic shunt and impaired ornithine cycle activity in cirrhosis lead to skeletal muscle depletion and impaired ammonia detoxification causing hepatic encephalopathy [1]. This condition may be aggravated by malnutrition [12]. We observed that the amino acid signature associated with MHE was characterized by a 24% reduction in BCAA and a 19.5% lower Fischer’s ratio, while the Gln/Glu ratio increased by 38.3% compared to those without MHE. Our study showed that oral nutritional supplementation for 12 weeks with either HMB or HP treatments increased plasma levels of AAA, Ala, and urea without increasing Gln or ammonia. Interestingly, only oral supplementation with HMB increased plasma levels of BCAA.

There is ample evidence of the profound effects of liver disease on amino acid metabolism. Pioneering studies had shown long ago that increased concentrations of methionine and AAA with decreased concentrations BCAA were associated with liver cirrhosis [34,35]. Here, we also found elevated Hyp in cirrhotic patients, formed by post-translational hydroxylation of proline and biomarkers of liver fibrosis [36]. Additionally, by using a multivariate (PCA) analysis we were able to include Arg, Asp, Ser, Lys, and Ala in the metabolic profile to distinguish healthy from malnourished cirrhotic patients.

Hyperammonemia is a consequence of portosystemic shunting and hepatocellular dysfunction, the skeletal muscle being the main system for ammonia detoxification in liver cirrhosis [2]. Hyperammonemia has been shown to be associated with the pathogenesis of complications related to cirrhosis, such as hepatic encephalopathy [37]. Beyond hyperammonemia, our results also agree with other metabolic routes previously described to be associated with HE. Thus, the decreased Fisher´s ratio may cause an imbalance in the synthesis of neurotransmitters [38] while increased Gln correlates with astrocyte swelling [11]. Reduced levels of taurine and Arg may indicate an impairment in the trans-sulphuration pathway which consumes hCys, a well-known risk factor for dementia [39,40,41]. Clinically, BCAA supplementation in liver cirrhosis have been associated with symptomatic improvement of hepatic encephalopathy [42,43]. Most studies indicate that administration of BCAA in cirrhosis has no effect or decreases ammonia [20]. However, Dam et al. observed a significant increase in arterial blood concentrations of ammonia and Gln flux from muscles in cirrhotic patients compared to healthy subjects after the intake of BCAA [44]. However, BCAA supplementation increased plasma BCAA levels to a lesser extent in cirrhosis than in healthy individuals. Holecek et al. described an increase in plasma and muscle levels of BCAA, Ala, and Gln in healthy rats orally supplemented with BCAA [21]. Interestingly, only oral supplementation with HMB increased plasma levels of BCAA although BCAA is also found in the High Protein ONS. We hypothesize that HMB is increasing plasma levels of BCAA via a direct effect on protein synthesis and inhibition of protein breakdown. Alternatively, HMB might also decrease gluconeogenesis [45] and consequently reduce the utilization of BCAA as gluconeogenic precursors.

HMB is a naturally occurring and metabolically active derivative of the BCAA leucine. Mounting evidence supports that ONS with HMB can increase muscle mass and strength and reduce muscle damage during resistance exercises [22,46,47,48], as well as prevent muscle loss in the elderly [49,50]. However, there are few clinical reports of the effects of ONS enriched with HMB on other muscle-wasting diseases [23] and specifically there are no data on the clinical impact of HMB in liver cirrhosis or its effects on plasma amino acid profiles. Holecek et al. conducted a study in healthy rats to examine the role of exogenous HMB on Leu and protein metabolism, and they observed an increase in plasma Leu levels as a result of a decrease in its clearance, as well as a decrease in plasma levels of Ala, Gln, and Glu [51].

We found several trends that can help focus future research. HMB supplementation increased fasting plasma levels of the essential amino acids Val, Leu, Phe, and Trp. We did not observe those changes in the HP group. It can be argued that the HMB group received the Ensure^®^ Plus Advance formula with a slightly higher dose of amino acids than Ensure^®^ Plus High Protein (HP treatment). However, the magnitude of the increases (27–35%) almost doubled the differences in the composition (13–20%). Both ONS increased the plasma levels of AAA (*p* = 0.003), Ala (*p* = 0.004) and urea (*p* = 0.048), without increasing the plasma levels of ammonia (*p* = 0.11) or Gln (*p* = 0.932). Fischer´s ratio increased significantly at the end of the study (*p* = 0.013) without differences between treatments, although an upward trend was observed in the HMB group. We also study the Gln/Glu ratio because an increased ratio has been associated with a deterioration of brain function [52] but no significant differences were found at the end of the trial for either of the two treatments (*p* = 0.31).

We found that the presence of MHE was associated with (1) reduced total plasma amino acid concentration, likely associated with an impaired nutritional status, (2) reduced BCAA and Fischer´s ratio, and (3) increased Gln/Glu ratio. This amino acid signature may be used to evaluate potential treatments to reduce the incidence of MHE in malnourished patients with liver cirrhosis. In this vein, our study showed that MHE events were nearly halved in individuals supplemented with HMB-enriched ONS after 12 weeks of treatment. Furthermore, HMB-enriched ONS increased Fischer´s ratio by increasing BCAA levels in a greater proportion than AAA levels, which according to previous results is associated with an improvement in the symptoms of hepatic encephalopathy [3].

Our study has several limitations, mainly derived from the complexity of amino acid metabolism in the MHE context. Studying plasma concentrations of amino acids can, at best, reflect only partial aspects of this complexity. We cannot rule out some other effects beyond the observed variation at the metabolomics level. Some HMB effects may occur at the gene expression level (reviewed in [45]) and a recent report has indicated that HMB is an epigenetic regulator in muscle progenitor cells in vitro [53]. The relatively small sample size has surely limited the power to find statistically significant associations. However, we find value in the overall description of these little-studied relationships. Taken together, the findings were consistent and this study will serve as an exploratory analysis for other potential linkages.

## 5. Conclusions

The main conclusion for this work is that HMB-enriched ONS increased the Fischer´s ratio in patients with liver cirrhosis and malnutrition without varying Gln or ammonia plasma levels, a protective amino acid profile that can help prevent MHE.

## Figures and Tables

**Table 1:** Plasma amino acid concentration in patients with cirrhosis compared to healthy controls.

	Cirrhosis	Healthy	*p*
	*n* = 43	*n* = 31	
Sex (men/women)	27 (62%)/16 (38%)	15 (48%)/16 (52%)	0.362
Age (years)	64.4 [55.3;68.6]	57.0 [47.5;66.5]	0.054
Amino acid (µM)			
phospho-Ser	3.10 [2.16;4.44]	12.2 [8.75;16.9]	**<0.001**
Taurine	64.1 [52.7;83.7]	39.8 [35.4;55.5]	**<0.001**
Asp	27.2 [21.9;37.0]	1.75 [1.27;2.51]	**<0.001**
Thr	132 [105;172]	120 [110;147]	0.429
Ser	151 [125;177]	92.0 [78.4;102]	**<0.001**
Asn	74.6 [58.3;83.2]	100 [77.9;112]	**<0.001**
Glu	156 [120;221]	30.3 [20.1;50.1]	**<0.001**
Gln	317 [249;393]	631 [557;685]	**<0.001**
Aminoadipic acid	3.67 [3.14;4.54]	0.73 [0.00;2.85]	**<0.001**
Gly	276 [228;322]	195 [169;221]	**<0.001**
Ala	305 [246;391]	333 [288;378]	0.354
Citrulline	42.3 [30.7;50.8]	36.2 [31.1;50.1]	0.629
Aminobutyric acid	15.2 [11.6;19.4]	14.7 [9.99;18.7]	0.600
Val	166 [128;221]	202 [190;255]	**0.010**
Cystine	1.77 [0.71;4.92]	46.2 [33.3;58.2]	**<0.001**
Met	34.1 [25.9;42.6]	17.2 [13.9;21.9]	**<0.001**
Ile	54.1 [47.2;66.5]	47.8 [40.1;59.5]	0.072
Leu	103 [83.6;123]	103 [89.3;121]	0.899
Tyr	104 [75.6;129]	56.4 [47.6;65.3]	**<0.001**
β-Ala	2.73 [1.25;4.34]	0.00 [0.00;0.00]	**<0.001**
Phe	89.7 [72.1;104]	44.7 [41.0;50.7]	**<0.001**
Ethanolamine	18.5 [13.9;23.4]	0.00 [0.00;0.00]	**<0.001**
Ornithine	93.0 [74.0;120]	77.6 [69.0;87.3]	**0.009**
Lys	163 [123;187]	173 [155;190]	0.159
1-Methylhistidine	11.9 [8.04;25.3]	10.3 [5.24;15.4]	0.060
His	89.3 [74.0;97.3]	57.6 [47.3;63.6]	**<0.001**
Trp	42.7 [30.4;52.8]	30.4 [25.0;37.7]	**0.015**
3-Methylhistidine	5.58 [3.94;8.07]	1.37 [0.00;3.40]	**<0.001**
Arg	120 [102;151]	54.1 [40.6;69.3]	**<0.001**
Hyp	16.4 [10.8;22.9]	9.87 [2.46;15.6]	**0.021**
Pro	205 [161;259]	174 [136;216]	0.092

Data are number (%) or median [interquartile range]. Bold in the column with *p*-values is to highlight statistical significance.

**Table 2:** Demographics and baseline characteristics.

	HMB Group (*n* = 22)	HP Group (*n* = 21)	*p*
Age (Years)	60.4 ± 8.61	61.4 ± 9.27	0.711
Sex (men/women)	14 (64%)/8 (36%)	13 (62%)/8 (38%)	1.000
Etiology *n* (%)			
Alcohol	17 (77.3%)	11 (52.4%)	0.624
HCV	2 (9.09%)	3 (14.3%)	
MAFLD	1 (4.55%)	3 (14.3%)	
Others	2 (9.09%)	4 (19%)	
Active alcoholism	4 (18.1%)	4 (19%)	0.683
Ascites	12 (54.5%)	9 (42.9%)	0.645
Refractory ascites	4 (18.3%)	0 (0%)	0.108
TIPS	1 (4.55%)	1 (4.76%)	1.000
Previous encephalopathy	2 (9.09%)	3 (14.3%)	0.664
MHE (PHES) *	8 (36.4%)	4 (19%)	0.355
Child-Pugh			0.398
Class A	9 (40.9%)	10 (47.6%)	
Class B	11 (50%)	11 (52.3%)	
Class C	2 (9.09%)	0 (0%)	
MELD	12.7 ± 5.31	13 ± 4.7	0.835
SGA			0.355
Class B	14 (63.6%)	17 (81.0%)	
Class C	8 (36.4%)	4 (19.0%)	
Ammonia (µM)	56.0 [40.0;83.0]	54.0 [39.8;78.0]	0.923

Data are number (%), mean ±SD or median [IQR]. HCV: Hepatitis C Virus, MAFLD: Metabolic Associated Fatty Liver Disease, TIPS: Transjugular intrahepatic portosystemic shunt, MHE: Minimal Hepatic Encephalopathy, PHES: Psychometric Hepatic Encephalopathy Score, MELD: Model for End stage Liver Disease, SGA: Subjective Global Assessment. * Diagnosis of MHE by PHES.

**Table 3:** Amino acid and ammonia levels for all subjects during follow-up according to treatment group.

Plasma Amino Acid (µM)	HMB Group			HP Group			*p_long_*	p_long*treatment_
	Baseline	6 wk.	12 wk.	Baseline	6 wk.	12 wk.		
Leu	92.4 [76.7;117]	120 [105;132]	117 [105;130]	117 [95.5;137]	116 [102;134]	118 [93.9;140]	**0.01**	**0.035**
Ile	53.3 [47.7;59.9]	63.1 [56.7;72.8]	61.9 [52.8;70.9]	62.6 [49.6;71.9]	57.9 [50.2;66.2]	56.3 [49.3;75.5]	0.532	0.084
Val	144 [109;191]	199 [180;245]	194 [161;210]	191 [160;253]	220 [173;244]	215 [167;237]	**0.004**	0.055
BCAA	300 [244;361]	382 [348;428]	376 [320;404]	370 [297;462]	385 [325;425]	380 [306;456]	**0.012**	**0.046**
Phe	81.5 [72.8;94.2]	118 [81,4;128]	111 [96.6;118]	102 [78.2;122]	115 [86.9;129]	102 [92.6;119]	**0.001**	0.057
Tyr	101 [73.1;129]	119 [74.0;150]	118 [80.5;137]	104 [91.5;130]	131 [110;158]	106 [98.3;119]	**0.001**	0.408
Trp	35.6 [30.8;45.6]	47.7 [39.4;59.6]	48.2 [34.2;61.8]	51.3 [33.2;67.2]	59.1 [46.4;62.2]	56.7 [40.8;64.8]	**0.012**	0.066
AAA	234 [188;265]	264 [196;342]	284 [226;310]	270 [190;324]	316 [229;344]	266 [247;304]	**0.003**	0.103
Fischer´s ratio	1.59 [1.32;2.43]	1.58 [1.37;2.11]	1.70 [1.39;2.29]	1.80 [1.43;2.21]	1.57 [1.25;1.84]	1.64 [1.39;2.03]	**0.013**	0.586
Glu	134 [118;184]	223 [170;302]	180 [142;235]	182 [151;269]	190 [118;252]	212 [178;249]	0.214	0.164
Gln	317 [224;390]	284 [227;348]	322 [215;344]	308 [262;372]	315 [264;400]	332 [275;381]	0.932	0.862
Gln/Glu ratio	2.16 [1.15;3.32]	1.78 [0.72;2.24]	1.69 [1.31;2.46]	1.63 [1.10;2.79]	2.07 [1.17;2.64]	1.52 [1.24;2.18]	0.31	0.272
Ala	267 [216;340]	371 [268;464]	360 [332;427]	331 [283;442]	392 [332;476]	391 [302;428]	**0.004**	0.093
Ammonia (µM)	56.0 [40.0;83.0]	59.0 [50.5;85.2]	67.0 [49.8;74.8]	54.0 [39.8;78.0]	62.0 [53.2;87.2]	67.0 [55.5;75.5]	0.11	0.689

Data are number median [IQR]. Wk.: weeks. BCAA: sum of Leu, Ile & Val. AAA: sum of Phe, Tyr, Trp. *p_long_*: *p*-value for the variation over time for the entire cohort. p_long*treatment_: *p*-value for treatment-specific longitudinal changes. Bold in the column with *p*-values is to highlight statistical significance.

**Table 4:** Association between plasma amino acid levels and minimal hepatic encephalopathy in all cirrhotic patients (MHE).

Plasma Amino Acid Concentration (µM)	MHE − *n* = 82	MHE + *n* = 26	*p*
Phospho-Ser	3.37 [2.46;4.33]	2.37 [1.83;3.93]	0.061
Taurine	76.0 [57.0;94.2]	56.6 [44.1;66.9]	**0.004**
Asp	36.2 [28.3;43.0]	20.2 [14.4;26.5]	**<0.001**
Thr	143 [117;175]	135 [119;174]	0.882
Ser	167 [146;184]	128 [103;160]	**<0.001**
Asn	73.6 [64.3;84.8]	78.0 [64.1;90.5]	0.402
Glu	209 [161;270]	135 [95.5;177]	**<0.001**
Gln	319 [242;382]	309 [287;419]	0.722
Aminoadipic acid	3.96 [2.98;4.77]	3.28 [2.65;4.09]	0.150
Gly	299 [266;331]	261 [230;283]	**0.002**
Ala	377 [311;468]	267 [246;353]	**<0.001**
Citrulline	44.7 [37.3;53.8]	47.3 [39.0;58.4]	0.327
Aminobutyric acid	15.5 [12.4;20.4]	14.7 [8.06;17.5]	0.121
Val	202 [171;246]	146 [114;181]	**<0.001**
Cystine	1.23 [0.00;3.38]	1.52 [0.71;4.95]	0.267
Met	36.1 [27.5;47.6]	37.5 [28.9;40.7]	0.840
Ile	62.1 [52.6;73.6]	48.5 [44.1;59.9]	**0.001**
Leu	118 [103;139]	91.2 [68.0;104]	**<0.001**
Tyr	113 [87.8;138]	112 [91.5;141]	0.735
β-Ala	3.85 [0.89;5.94]	3.62 [1.21;5.00]	0.622
Phe	105 [88.9;125]	86.7 [76.7;113]	0.045
Ethanolamine	17.2 [13.8;21.2]	20.8 [16.7;28.5]	0.121
Ornithine	105 [84.6;131]	98.1 [81.8;109]	0.241
Lys	179 [140;201]	147 [133;182]	0.098
1-Methylhistidine	12.4 [8.19;23.0]	11.6 [6.24;22.9]	0.482
His	89.0 [77.8;99.0]	87.1 [76.2;92.6]	0.216
Trp	51.9 [38.2;63.4]	45.4 [27.3;56.9]	0.089
3-Methylhistidine	7.00 [4.31;11.5]	6.86 [5.14;9.01]	0.758
Arg	133 [114;151]	113 [85.3;144]	**0.032**
Hyp	17.2 [10.5;23.2]	16.8 [13.4;24.8]	0.639
Pro	232 [178;278]	239 [174;329]	0.591
BCAA	383 [325;457]	289 [220;336]	**<0.001**
AAA	269 [220;320]	237 [193;297]	0.211
Fischer ratio	1.80 [1.38;2.42]	1.45 [1.18;1.60]	**0.001**
Gln/Glu ratio	1.72 [1.11;2.19]	2.38 [1.48;3.93]	**0.022**

Data are number (%) or median [interquartile range]. MHE: minimal hepatic encephalopathy. −: absent, +: present. BCAA: branched chain amino acids, AAA: aromatic amino acids. Bold in the column with *p*-values is to highlight statistical significance.

## Data Availability

The data presented in this study are available on request from the corresponding author with prior authorization of our Ethical Committee that can be obtained at https://www.iacs.es/investigacion/comite-de-etica-de-la-investigacion-de-aragon-ceica/ceica-evaluaciones-y-otras-presentaciones accessed on 22 October 2021.

## Supplementary Materials

The following are available online at https://www.mdpi.com/article/10.3390/nu13113764/s1, Supplementary Table S1. Amino acids nutritional content of each oral supplement, Supplementary Table S2. Amino acid levels and analytical parameters at baseline, Supplementary Table S3. Amino acid levels (median) during follow-up.
