# Supplementary material for: Amino Acid Profile in Malnourished Patients with Liver Cirrhosis and Its Modification with Oral Nutritional Supplements: Implications on Minimal Hepatic Encephalopathy
Source: Nutrients. 2021 Oct 25;13(11):3764. doi: 10.3390/nu13113764 (PMC8617874; doi:10.3390/nu13113764)
Supplement: Supplementary file 1 [file nutrients-13-03764-s001.zip › nutrients-1425972-supplementary.pdf]

Supplemental Table S1. Amino acids nutritional content of each oral supplement

Amino acids (g/100ml)	Ensure® Plus Advance	Ensure® Plus High Protein
**Essential**		
Histidine	0.22	0.20
Isoleucine	0.45	0.37
Leucine	0.81	0.70
Lysine	0.68	0.57
Methionine	0.21	0.19
Phenylalanine	0.43	0.38
Threonine	0.41	0.33
Tryptophan	0.12	0.10
Valine	0.52	0.46
Arginine	0.35	0.32
**Non-essential**		
Alanine	0.31	0.25
Aspartic acid	0.34	0.27
Cystine	0.07	0.04
Glutamic acid	0.95	0.84
Glycine	0.20	0.17
Proline	0.82	0.75
Serine	0.49	0.44
Tyrosine	0.42	0.39
Asparagine	0.40	0.34
Glutamine	0.90	0.79

Supplemental Table S2. Amino acid levels and analytical parameters at baseline

Plasma amino acid concentration (µM)	HMB Group N=22	HP Group N=21	p
**phospho-Ser**	2.50 [2.05;3.90]	3.20 [2.18;4.31]	0.474
**Taurine**	64.1 [53.4;81.3]	72.7 [51.2;88.3]	0.955
**Asp**	26.0 [21.4;35.5]	30.3 [24.0;39.5]	0.320
**Thr**	132 [102;172]	133 [107;166]	0.696
**Ser**	147 [129;171]	161 [108;180]	0.566
**Asn**	72.9 [59.2;80.1]	74.6 [57.1;85.0]	0.696
**Glu**	134 [118;184]	182 [151;269]	0.152
**Gln**	317 [224;390]	308 [262;372]	0.958
**Aminoadipic acid**	3.42 [2.92;4.33]	4.08 [3.55;4.38]	0.241
**Gly**	275 [258;295]	277 [227;322]	0.953
**Ala**	267 [216;340]	331 [283;442]	0.068
**Citrulline**	41.0 [30.1;50.2]	42.5 [37.2;50.8]	0.726
**Aminobutyric acid**	14.7 [9.50;19.3]	15.4 [13.6;21.8]	0.241
**Val**	144 [109;191]	191 [160;253]	0.051
**Cystine**	1.64 [0.59;2.77]	1.54 [0.78;5.19]	0.412
**Met**	30.8 [25.8;45.9]	36.8 [27.8;42.6]	0.389
**Ile**	53.3 [47.7;59.9]	62.6 [49.6;71.9]	0.163
**Leu**	92.4 [76.7;117]	117 [95.5;137]	**0.047**
**Tyr**	101 [73.1;129]	104 [91.5;130]	0.554
**β-Ala**	3.49 [1.57;4.50]	2.60 [1.09;4.53]	0.666
**Phe**	81.5 [72.8;94.2]	102 [78.2;122]	0.070
**Ethanolamine**	15.5 [11.7;22.9]	20.1 [15.9;22.5]	0.141
**Ornithine**	99.0 [71.3;110]	89.2 [78.8;131]	0.678
**Lys**	150 [113;170]	180 [132;195]	0.141
**1-Methylhistidine**	12.3 [8.41;25.3]	11.7 [7.48;25.3]	0.626
**His**	87.1 [74.9;92.7]	90.3 [80.1;100]	0.209
**Trp**	35.6 [30.8;45.6]	51.3 [33.2;67.2]	**0.046**
**3-Methylhistidine**	5.38 [4.17;8.66]	6.51 [3.96;8.95]	0.882
**Arg**	114 [103;150]	133 [95.3;152]	0.566
**Hyp**	17.0 [11.8;22.6]	15.1 [9.42;23.2]	0.687
**Pro**	228 [153;258]	237 [170;278]	0.414
**BCAA**	300 [244;361]	370 [297;462]	0.060
**AAA**	234 [188;265]	270 [190;324]	0.110
**Fischer ratio**	1.59 [1.32;2.43]	1.80 [1.43;2.21]	0.514
**Gln/Glu ratio**	2.16 [1.15;3.32]	1.63 [1.10;2.79]	0.267

HMB: ß-Hydroxy-ß-methylbutyrate, HP: High Protein, BCAA: branched chain amino acids, AAA: aromatic amino acids.

Supplemental Table S3. Amino acid levels (median) during follow-up

	HMB Group			HP Group		
	**Baseline**	**6 weeks**	**12 weeks**	**Baseline**	**6 weeks**	**12 weeks**
**Phospho-Ser**	2.50 [2.05;3.90]	3.30 [2.60;4.28]	3.26 [1.92;3.64]	3.20 [2.18;4.31]	3.52 [2.40;4.16]	3.23 [2.36;4.86]
**Taurine**	64.1 [53.4;81.3]	57.1 [52.4;77.2]	79.0 [55.8;88.7]	72.7 [51.2;88.3]	75.0 [66.1;113]	85.0 [65.2;95.6]
**Asp**	26.0 [21.4;35.5]	37.1 [19.8;44.5]	32.0 [24.8;39.1]	30.3 [24.0;39.5]	40.2 [27.4;44.3]	35.8 [28.6;41.8]
**Thr**	132 [102;172]	120 [106;182]	144 [122;163]	133 [107;166]	156 [132;163]	171 [136;193]
**Ser**	147 [129;171]	152 [138;185]	166 [146;174]	161 [108;180]	165 [150;185]	171 [140;182]
**Asn**	72.9 [59.2;80.1]	66.7 [60.5;88.9]	70.2 [67.7;84.9]	74.6 [57.1;85.0]	77.2 [71.4;91.1]	82.0 [67.7;91.2]
**Glu**	134 [118;184]	223 [170;302]	180 [142;235]	182 [151;269]	190 [118;252]	212 [178;249]
**Gln**	317 [224;390]	284 [227;348]	322 [215;344]	308 [262;372]	315 [264;400]	332 [275;381]
**Aminoadipic acid**	3.42 [2.92;4.33]	3.56 [2.20;3.84]	4.60 [3.34;5.75]	4.08 [3.55;4.38]	3.90 [1.84;4.63]	4.44 [2.73;5.05]
**Gly**	275 [258;295]	280 [263;334]	300 [263;330]	277 [227;322]	292 [259;308]	289 [270;316]
**Ala**	267 [216;340]	371 [268;464]	360 [332;427]	331 [283;442]	392 [332;476]	391 [302;428]
**Citrulline**	41.0 [30.1;50.2]	43.5 [32.7;49.1]	44.0 [38.0;51.4]	42.5 [37.2;50.8]	49.7 [45.1;60.2]	50.7 [44.7;61.6]
**Aminobutyric acid**	14.7 [9.50;19.3]	16.0 [11.8;21.1]	16.3 [12.4;19.7]	15.4 [13.6;21.8]	14.1 [10.9;23.5]	14.8 [12.0;19.0]
**Val**	144 [109;191]	199 [180;245]	194 [161;210]	191 [160;253]	220 [173;244]	215 [167;237]
**Cystine**	1.64 [0.59;2.77]	2.30 [0.99;4.53]	1.25 [0.84;3.68]	1.54 [0.78;5.19]	1.03 [0.00;2.24]	0.65 [0.00;2.97]
**Met**	30.8 [25.8;45.9]	32.5 [24.6;50.2]	36.5 [27.4;48.6]	36.8 [27.8;42.6]	41.5 [36.3;49.3]	35.5 [33.2;46.0]
**Ile**	53.3 [47.7;59.9]	63.1 [56.7;72.8]	61.9 [52.8;70.9]	62.6 [49.6;71.9]	57.9 [50.2;66.2]	56.3 [49.3;75.5]

**Leu**	92.4 [76.7;117]	120 [105;132]	117 [105;130]	117 [95.5;137]	116 [102;134]	118 [93.9;140]
**Tyr**	101 [73.1;129]	119 [74.0;150]	118 [80.5;137]	104 [91.5;130]	131 [110;158]	106 [98.3;119]
**ß-Ala**	3.49 [1.57;4.50]	3.85 [2.89;6.44]	4.29 [1.23;7.28]	2.60 [1.09;4.53]	3.65 [0.36;5.50]	5.06 [0.00;6.49]
**Phe**	81.5 [72.8;94.2]	118 [81.4;128]	111 [96.6;118]	102 [78.2;122]	115 [86.7;129]	102 [92.6;119]
**Ethanolamine**	15.5 [11.7;22.9]	15.1 [13.1;19.6]	15.8 [13.9;19.6]	20.1 [15.9;22.5]	19.2 [16.8;30.6]	20.9 [16.3;24.5]
**Ornithine**	99.0 [71.3;110]	113 [86.0;144]	105 [95.3;115]	89.2 [78.8;131]	98.7 [84.6;125]	108 [95.2;121]
**Lys**	150 [113;170]	171 [144;195]	173 [133;208]	180 [132;195]	181 [153;201]	179 [143;214]
**1.Methylhistidine**	12.3 [8.41;25.3]	9.38 [6.14;24.6]	11.8 [7.34;17.5]	11.7 [7.48;25.3]	15.0 [10.1;20.5]	16.9 [8.55;20.8]
**His**	87.1 [74.9;92.7]	82.2 [71.4;96.0]	83.7 [76.3;94.3]	90.3 [80.1;100]	89.0 [83.5;99.0]	87.5 [77.8;101]
**Trp**	35.6 [30.8;45.6]	47.7 [39.4;59.6]	48.2 [34.2;61.8]	51.3 [33.2;67.2]	59.1 [44.6;62.2]	56.7 [40.8;64.8]
**3-Methylhistidine**	5.38 [4.17;8.66]	8.57 [6.75;11.4]	8.80 [5.41;12.3]	6.51 [3.96;8.95]	7.15 [3.67;9.27]	6.61 [5.28;9.71]
**Arg**	114 [103;150]	134 [109;153]	133 [116;155]	133 [95.3;152]	130 [111;152]	129 [116;147]
**Hyp**	17.1 [11.8;22.6]	18.3 [15.1;24.7]	17.4 [13.9;27.1]	15.1 [9.42;23.2]	15.8 [9.40;20.7]	16.7 [11.0;24.5]
**Pro**	228 [153;258]	241 [182;269]	219 [198;242]	237 [170;278]	235 [217;309]	214 [170;289]
